# What is a fire resilient landscape? Towards an integrated definition

**DOI:** 10.1007/s13280-023-01891-8

**Published:** 2023-06-30

**Authors:** Fiona E. Newman Thacker, Marc Castellnou Ribau, Harm Bartholomeus, Cathelijne R. Stoof

**Affiliations:** 1 Soil, Physics and Land Management Group, Wageningen University & Research, 6708 PB Wageningen, The Netherlands; 2 Catalan Fire Service, Bombers GRAF, Parc de Bombers de Cerdanyola del Vallès, Av. De Serragalliners, 08193 Cerdanyola del Vallès, Spain; 3 Laboratory of Geo-Information Science and Remote Sensing, Wageningen University & Research, 6708 PB Wageningen, The Netherlands

**Keywords:** Resilient landscapes, Socio-ecological system, Transformative resilience, Wildfire

## Abstract

The concept of fire resilience has become increasingly relevant as society looks to understand and respond to recent wildfire events. In particular, the idea of a ‘fire resilient landscape’ is one which has been utilised to explore how society can coexist with wildfires. However, the concept of fire resilient landscapes has often been approached in silos, either from an environmental or social perspective; no integrated definition exists. Based on a synthesis of literature and a survey of scientists and practitioners, we propose to define a fire resilient landscape as ‘a socio-ecological system that accepts the presence of fire, whilst preventing significant losses through landscape management, community engagement and effective recovery.’ This common definition could help guide policy surrounding fire resilient landscapes, and exemplify how such landscapes could be initiated in practice. We explore the applicability of the proposed definition in both Mediterranean and temperate Europe.

**Supplementary Information:**

The online version contains supplementary material available at 10.1007/s13280-023-01891-8.

## Introduction

Wildfires are a global phenomenon which have been part of many ecosystems for thousands of years. Fire has also been used anthropogenically as an ancestral tool to shape landscapes, resulting in particular fire-dependent ecosystems, such as the UK’s heather moorland (Dodgshon and Almered 2006; Yallop et al. 2006) and the Pyrenees grassland (Múgica et al. 2021). However, in recent decades, extreme fires have become more common, resulting in significant damage (Tedim and Leone 2017; Dupuy et al. 2020). In addition, wildfires are becoming increasingly prevalent and severe in temperate regions (Belcher et al. 2021; Stoof and Kettridge 2022). With projected climate changes indicating that vulnerability to wildfire is likely to get worse, improved understanding of these events is important (Pueyo 2007; Goss et al. 2020). In particular, it is vital to further move away from singularly reactive approaches towards more proactive and preventative measures. The concept of a fire resilient landscape has arisen from the recognition that the general trend in many countries to supress and exclude fire from the landscape has conversely led to increased wildfire risk (Kauffman 2004). When combined with the difficulties presented in managing forested lands, particularly in areas experiencing high levels of rural depopulation, these factors have contributed to some of the most disastrous fire seasons on record (Moreira et al. 2011; Turco et al. 2014; Collins et al. 2019; Higuera and Abatzoglou 2021). Recognising that current strategies are not working, perceptions surrounding wildfires are beginning to shift away from just fire suppression—and from this the notion that societies should begin to ‘live with fire’ is slowly gaining traction (Tedim and Leone 2017; McWethy et al. 2019; Stoof and Kettridge 2022).

The idea of a fire resilient landscape has grown in an attempt to understand the complicated relationship between society, nature and fire (Smith et al. 2016). However, the definition of such a landscape remains undetermined, with no inherent guidelines or blueprint (Wunder et al. 2021). Terminology surrounding wildfire events is often contentious, as exemplified by Keeley (2009) who identifies the potential issues caused by the malleable use of fire intensity, fire severity and burn severity. In particular, landscape resilience in the context of wildfire is a term that is being increasingly utilised, but remains nebulous and often concentrated on environmental characteristics (Moritz et al. 2011; Magalhães et al. 2021).

This study engaged with the fire community to explore the intrinsic values connected with a fire resilient landscape; those which are consistently associated with the term. In this study, the fire community involved professionals working in both practice, e.g. land management, firefighting and science, e.g. academia, research institutes, alongside students. This analysis approached landscapes from a socio-ecological perspective, whereby social and ecological systems are coupled (Spies et al. 2014; Fernández-Blanco et al. 2022). We reviewed current research on fire resilient landscapes, and conducted a survey to elucidate the common themes of such landscapes, alongside proposing an overall definition. We then applied these common themes and definition to two case studies in Europe to illustrate their applicability.

## Resilience in the firescape: Literary overview

With the recognition that the dominant, reactive approach to wildfire events are making extreme events more common (Castellnou et al. 2019), resilience has become increasingly prominent as the fire community works to move beyond suppressive techniques.

**Fig. 1:**
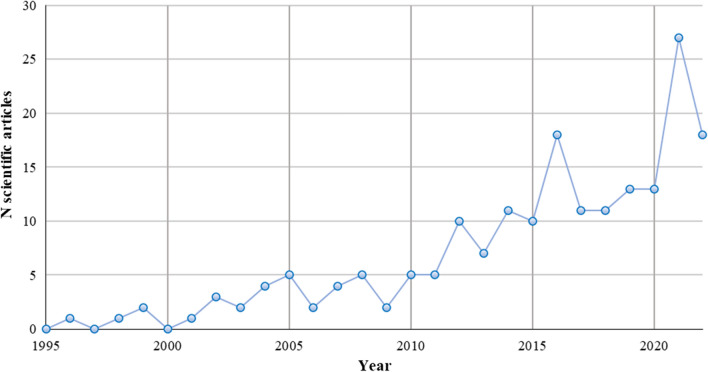
Increasing attention to fire resilient landscapes in the scientific literature: the number of scientific articles linked to fire resilience available on Web of Science and Scopus from 1995 to 2022. The search words ‘Fire’ AND ‘Resilient’ AND ‘Landscape’ were used to extract papers which contained these words in the title, abstract and/or keywords. The terms were searched for separately, and returned similar words. For example ‘fire’ also returned ‘wildfire’. The results were manually filtered to remove unrelated research. See supplementary information for more information

Figure 1 illustrates the rise in popularity surrounding fire resilient landscapes as a research topic.

Resilience as a concept for understanding ecological systems was first defined by Holling (1973, p. 17) as “a measure of the ability of systems to absorb changes of state variables, driving variables and parameters and still exist”. This definition has also been explored and applied to systems beyond ecology. For example, Gunderson and Holling (2002) develop the concept of panarchy to apply resilience to both social and ecological systems in an integrated manner. Adger (2000) also highlights the important feedbacks and dependencies between social and ecological resilience in the context of resource management. Brown (2014) further emphasises the social dimension of resilience, concluding there has been advances in the integration of social factors in the context of global environmental change, but further assimilation is still needed. Resilience has also evolved from Holling’s (1973) initial definition. The concept of adaptive resilience emerged to understand how characteristics of social systems can be altered to adapt to new conditions, whilst transformative resilience depicts the way systems may ‘bounce forward’ in circumstances where returning to previous conditions is undesirable or impossible (Schoennagel et al. 2017; McWethy et al. 2019; Asadzadeh et al. 2022). Below we summarise how resilience has been explored in the context of wildfire events and highlight existing knowledge gaps.

Despite aforementioned research in resilience studies highlighting the importance of social and ecological integration, the typical way of tackling wildfire events is to look for context-specific resilience—seeking solutions in silos (Smith et al. 2016; Belcher et al. 2021). Often, only ecological characteristics of a landscape are considered, as evidenced by the definition for landscape fire resilience offered by Magalhães et al. (2021, p. 3): “the capacity of a landscape in absorbing the disturbance caused by rural fires without losing its function, structure and identity and ultimately weakening fire frequency and intensity or magnitude”. Adámek et al. (2016) also focusses on ecological landscape resilience, exploring the resilience of central European pine forests to wildfire, concluding this landscape is resilient if a certain fire frequency—once every 200 years—is upheld. Whilst the definition provided by Magalhães et al. (2021) and the concept of ecological resilience can be useful, it is also essential to recognise the human dimension of landscapes and community resilience. For example, community resilience is investigated by Scalingi (2020), examining how a holistic, multi-community method could be designed for residents in fire-prone areas of California. Vallianou et al. (2010) also concentrates on community resilience, whereby emotions and place attachment are explored to understand how post-trauma recovery is linked to resilience.

Examining each component of a landscape in isolation can allow deeper insights to be made within a specific context. However, wildfire is a ‘wicked problem’—one which is inherently difficult to solve and offers no singular solution (Rittel and Webber 1973). Consequently, a line of research has moved towards a more holistic approach, recognising that the connections between social and ecosystem resilience are important when considering fire resilient landscapes. Wunder et al. (2021) recognises wildfire as a wicked problem and looks towards a ‘theory of change’ for Mediterranean landscapes, suggesting that it is essential to include socio-economic factors when studying or structuring a fire resilient landscape. Leone et al. (2020) introduced the idea of a fire smart territory in an effort to empirically frame the concept of living with fire, approaching wildfires with a shared governance system—whereby communities are empowered with knowledge on resilience, whilst being able to access state-provided professional resources. Within this system there are two main scales of resilience; (1) homes and assets and (2) natural landscapes/forests, combining areas which are often addressed in isolation. Moritz et al. (2014) also aimed to understand wildfire resilience in a coupled system. By looking at the impact of fire in each individual system (social and ecological), and where they coincide at the wildland urban interface, this study emphasises that the presence of ‘good’ fire is essential for many ecosystems to be fire resilient. Smith et al. (2016) concludes similarly, stating that low intensity fires must be welcomed into the landscape alongside policy, education and knowledge sharing systems.

Within Smith et al. (2016) the phrase ‘firescape’, originally proposed by Wood et al. (2011), is expanded upon to emphasise the need to develop an integrated perspective of wildfires, looking to interweave wildfire events into every aspect of the social and physical landscape. However, it has also been highlighted that interweaving 'firescape' policies into landscapes is highly regional and dependent on social conditions, and that whilst the aim of a fire resilient landscape may remain the same, the pathways to achieving this can differ through place-based situations (Jakes et al. 2007; Paveglio et al. 2015; Uyttewaal et al. 2023).

What is clear from this overview of relevant literature is that the concept of a fire resilient landscape is complex and malleable, but that both ecological and social aspects are at its core. Therefore, the ecologically-focused definition proposed by Magalhães et al. (2021) is incomplete, as it lacks the cohesion of social processes inherent to fire. As such, the concept of a fire resilient landscape remains to be defined in an integrated way.

## Methodology

The aim of this study was to develop a definition of a fire resilient landscape by utilising a range of perspectives to derive what values can be found consistently amongst the targeted groups. An online survey was chosen due to greater geographical scope offered by this methodology in comparison to other qualitative data techniques such as interviews. Surveys have been commonly utilised in wildfire research to gather opinions on topics such as fuel management (McCaffrey et al. 2008; Fischer et al. 2014), firefighter safety (Flores and Haire 2022) and household preparedness for wildfires (Prior and Eriksen 2013). However, surveys have not been used to explore concepts around fire resilient landscapes, with most research developing theoretical frameworks instead (Smith et al. 2016; Schoennagel et al. 2017; Tedim and Leone 2017). Therefore, the use of survey methodology can address a knowledge gap as it can capture views from the wider fire community, going beyond merely the work published in the scientific literature.

Respondents were asked:

What does a fire resilient landscape mean to you? Please consider all your knowledge and experience.

The following question, alongside optional sharing of their country of work and job title, was used to explore participants perceptions of a fire resilient landscape. We sent the survey to two groups:

1. professionals working in fire including practitioners (fire managers, policy makers, etc.) and academics. The survey was distributed initially through the Flamework WhatsApp group containing 70 members of professional fire community across the world, who we invited to share the survey with colleagues.
2. pyrogeography students from Wageningen University, The Netherlands, enrolled in six different master programs spanning environmental and social sciences. 32 students were involved from 4 different countries. The course covered integrated fire management and the inter- and transdisciplinary context of fire; students were not taught directly about landscape resilience.

The sampling was largely determined by pre-existing networks available to the authors, with the aim to target a diversity of people working and learning with fire. 82 responses were recorded across both groups from 13 different countries; including traditional fire-prone countries (e.g. USA and Spain) alongside areas where wildfire is categorised as an emerging risk (e.g. UK, the Netherlands). Respondents reflected fire and land management (*n* = 25), research (*n* = 22) and unidentified (*n* = 35). Having perspectives from different backgrounds, as indicated in these two groups, is likely to be helpful in deriving a definition broad enough for inclusion but specific enough for utility.

Survey results were analysed qualitatively using Braun and Clarke’s (2006) thematic analysis, to produce common themes of a fire resilient landscape from the resulting codes. For details on the methods used, please refer to the supplementary information. The responses were also classified into three categories according to their overall focus.

- Environmental—focussing mainly on the physical environment and ecological characteristics.
- Social—focussing on communities and anthropological factors.
- Integrated—addressing both elements around the physical environment alongside social factors.

The responses were also linked to either working within practice, science or as a student, based on information given by the respondents.

## Defining a fire resilient landscape: five common themes

**Fig. 2:**
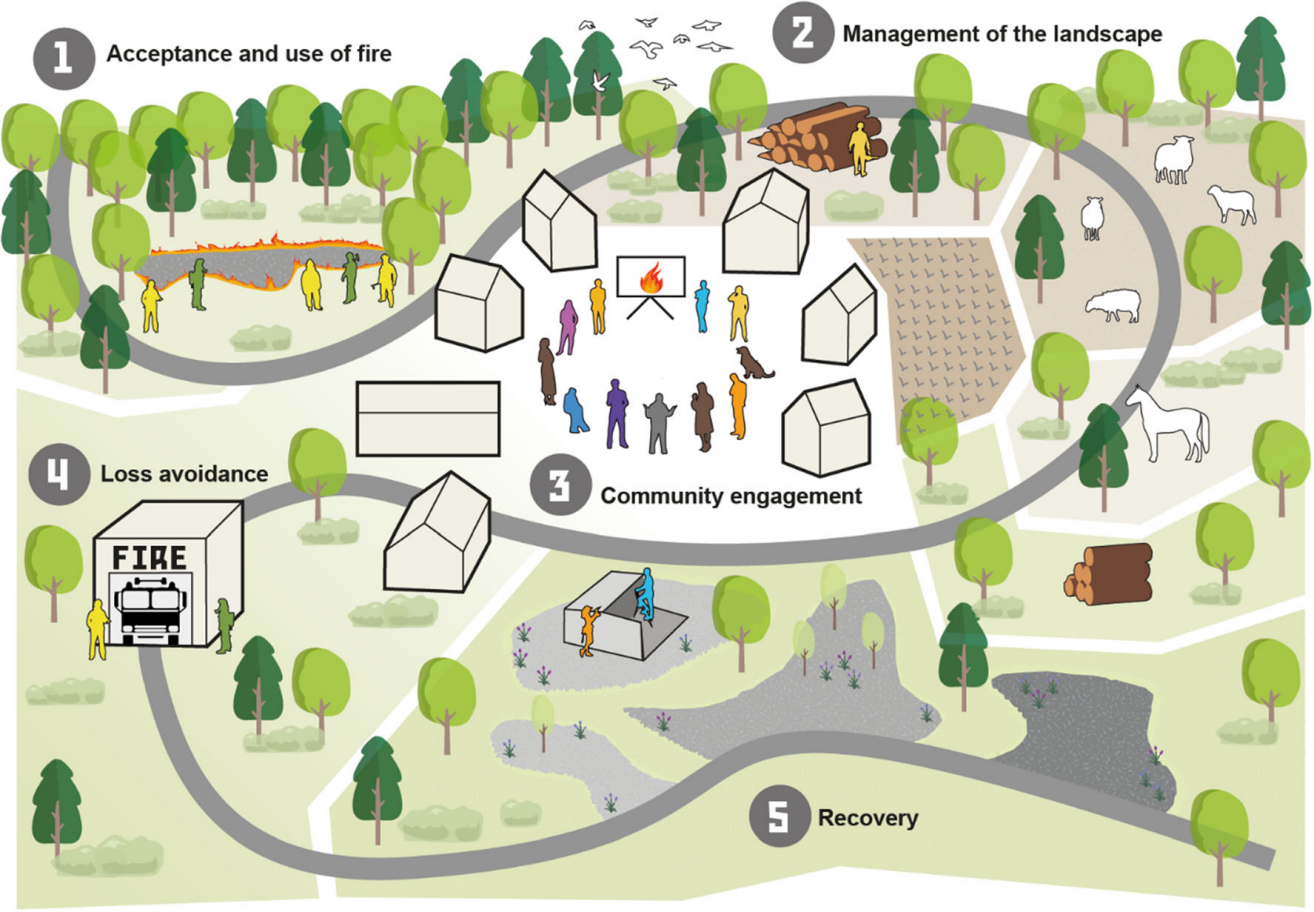
The common themes of a fire resilient landscape: acceptance and use of fire, management of the landscape, community engagement, loss avoidance and recovery

Thematic analysis of the survey responses revealed five common themes: acceptance and use of fire, management of the landscape, community engagement, loss avoidance; and recovery (Fig. 2):

1. Acceptance and use of fire: Participants frequently highlighted the presence of fire as a natural process (subtheme 1.1) as a pivotal element of a fire resilient landscape. As acknowledged in previous research, if fire is treated as an anomaly to be extinguished immediately, the ‘wildfire paradox’ takes control, and resilience to future events is decreased (Castellnou et al. 2019). The elimination of landscape fire, if previously it would occur naturally or has been a historical element of the landscape through anthropogenic use, also has cascading impacts on species diversity and landscape heterogeneity (Kauffman 2004). Respondents highlighted that using fire itself is also a strong factor in enhancing resilience, emphasised with the inclusion of prescribed burning (subtheme 1.2) as an important technique for reducing fuel loads and encouraging positive feedbacks (Moreira et al. 2011; Alcasena et al. 2018; Duane et al. 2019).
2. Management of the landscape: Respondents consistently acknowledged that, in many areas, an unmanaged landscape is unlikely to be a resilient one. They emphasised the importance of a heterogenous landscape with diverse species and a range of land uses. A mosaic landscape (subtheme 2.1), such as one where agriculture and forest are dispersed in parcels (Aquilué et al. 2020), or where there is diversity in the age and species of present vegetation, was consistently indicated as significant in building resilience. Mosaic landscapes are known to decrease fire rate of spread and offer safer areas for suppression (Alcasena et al. 2019). These landscapes can be enhanced by fuel management (subtheme 2.2), such as silvopasture, agroforestry and prescribed burning (Salis et al. 2018; North et al. 2021).
3. Community engagement: Responses indicated that anthropogenic landscape elements must be also resilient, through engagement with communities. Knowledge sharing, collaboration and education (subtheme 3.1) was sometimes deemed essential by respondents. Knowledge exchanges between fire services and communities can help residents understand wildfires and which preventative actions (subtheme 3.2) can be taken to mitigate potentially negative effects (Alcasena et al. 2019). Example of this include the FireWise program in the US and the UK (NFPA 2009; Berry et al. 2016; Dorset and Wiltshire Fire and Rescue 2019). Vice versa, local/indigenous place-based knowledge can help fire managers by confirming, for example, good access points or historical fire regimes (Smith et al. 2016). For this to be successful, respondents stressed that trust (subtheme 3.3) between the relevant parties is important.
4. Loss avoidance: A number of responses recognised that a resilient landscape is one where wildfire events do not turn into wildfire disasters, characterised by an absence of long-lasting damage to social infrastructure and ecosystems (subtheme 4.1). The importance of a knowledgeable and trained fire fighting force (subtheme 4.2) was identified as a key factor in loss avoidance due to their ability to provide strategic suppression. Teams that can work with fire to achieve the best outcome for the physical environment and communities involved, but also understand the need for good fire in the landscape, can be critical for resilience.
5. Recovery: Participants finally highlighted that a landscape should fulfil the original definition of resilience (Holling 1973), that it should be able to absorb shocks and return to a stable state (subtheme 5.1)**.** Multiple respondents highlighted that this stable state is not necessarily a mirror of the landscape prior to the fire, but may include changes depending on the outcome and response to the event. Recovery considers both the vegetation and ecological characteristics of a landscape, alongside the communities within it. Results also showed that fire-adapted species are often perceived as essential for a landscape to have capacity to recover (subtheme 5.2).

These common themes and subsequent subthemes show that a fire resilient landscape must address environmental and social processes, exemplifying the value in a holistic approach. This integrated view has gained traction but is not yet commonplace, illustrated by the breakdown of responses according to group and categories depicted in Table 1.

**Table 1:** The breakdown of survey responses according to the background of the respondent and the overall theme of the response

	Environmental	Social	Integrated
Science	*13*	*0*	*9*
Practice	*16*	*0*	*9*
Student	*6*	*1*	*25*
No data	*3*	*0*	*0*
Sum	*38*	*1*	*43*

Approximately half of survey answers covered both environmental and social considerations, but in both the ‘science’ and ‘practice’ group, environmental factors were more commonly considered. Interestingly, the students provided the most integrated responses, highlighting the value in teaching about wildfires from an integrated perspective.

Based on the themes we propose the following definition:

A fire resilient landscape is a socio-ecological system that accepts the presence of fire, whilst preventing significant losses through landscape management, community engagement and effective recovery.

This definition acknowledges the interconnections between social and ecosystem processes, yet is flexible enough to be used in a diversity of regions/fire regimes: all landscapes will have all themes represented, but the balance between the themes can vary. For example, in the rural urban interface community engagement (Theme 3) will require stronger emphasis than in a remote region. Likewise, in a boreal forest the acceptance of fire (Theme 1) is likely more relevant than the creation of a patchwork agricultural mosaic (Theme 2), owing to the unfeasibility of drastically changing boreal forest (Kharuk et al. 2021). As such, the proportional importance of each theme will differ according to the landscape in question. When applying the resulting themes and definition, it is important to recognise that the responses may not be a perfect representation of how all groups perceive fire resilient landscapes, due to an underrepresentation of certain regions, such as Africa and Asia. These regions remain underrepresented in scientific wildfire literature in general, and so further work could be done to explore the suggested concepts in these areas.

## Fire resilient landscapes in practice

The common themes of a fire resilient landscape apply to a multitude of environments and spatio-temporal scales, but also on the scale of an individual fire and within all phases of the fire management cycle. Here, we illustrate how such application can work in practice, using two examples from Mediterranean and temperate Europe based on the authors familiarity with regions and actors involved. These case studies include both an individual fire event, and an area that is engaging with proactive fire management.

### The Roses wildfire, Spain

The Roses wildfire ignited on 21st February 2022 in Catalonia, a region where wildfires are a regular occurrence and are an inherent part of the landscape, but which still struggles with the impacts of such events (Alcasena et al. 2019). The 400-hectare fire burned predominantly within the Parc Natural del Cap de Creus (10780 ha total). The vast majority (93%) of the area burnt consisted of shrub and grasslands, with the rest consisting of cultivated areas (0.5%) and wooded areas (7.5%). This is typical of Catalonia, where fires predominantly occur in shrubland (Moreira et al. 2011). Shrubland species (e.g. mastic trees, junipers and heather) are also the most common vegetation type in the national park, whilst tree species such as the Aleppo pine (*Pinus Halepensis*) and Cork oaks (*Quercus Suber*) are present but less widespread.

Whilst February is outside the normal fire season (Alcasena et al. 2019), large fires are becoming more common in ‘off season’ months due to recurring winter droughts, associated with climatic changes. Before the fire, fuel management in the park included the introduction of cattle grazing. Grazing (subtheme 2.1) was initiated to open the landscape and introduce vegetation heterogeneity; landscape management (Theme 2). Whilst the predominant aim was to improve biodiversity within the park, managers had previously discussed with the GRAF (Catalan Support Group for Forest Interventions) firefighting team about fire risk, building trust through collaboration (Theme 3). Acting on the advice and working alongside GRAF, park managers were able to reduce fire risk by including fire disturbance management within landscape planning (subtheme 3.2).

Alongside land managers, the fire service had also fostered relations with private land owners (subtheme 3.1) and discussed previous wildfires with them. Once the Roses fire ignited, a vineyard’s mosaic landscape provided a safe space for firefighters to work from. The vineyards also offered a safety buffer for a nearby urban interface, allowing firefighters to prioritise other flanks of the fire without high risk to the inhabitants. The collaboration with the landowner meant that she remained positive with the outcome of the fire, despite some heat damage to the vineyard. This example highlights the possible mutual benefit for both fire managers and community members—the firefighters were able to stay safe, whilst the landowner trusted the fire service to protect her property and land against substantial loss (subthemes 3.3, 4.2).

Although the Roses fire had the potential to progress into a high impact event due to drought conditions and the close proximity of tourist areas, overall perceptions of the fire were positive (subtheme 1.2). Prior to the fire ignition, the work done to integrate fire service knowledge of fire management into landscape planning (Theme 2) ensured fire severity and rate of spread remained low and no great losses were sustained (Theme 4). The knowledge and experience of the firefighters meant that the fire could be held at a low intensity, burning within particular boundaries and clearing shrubs and grasses (subtheme 4.1). Whilst these species are fire resilient (subtheme 5.2) and will therefore regrow easily, the initial reduction of fuels offers positive feedbacks. The newly burnt plot enhances the future resilience of the area—if another fire were to occur here in more extreme climatic conditions (higher temperatures, high wind speeds), the burnt patches would provide opportunities for safer suppression, alongside acting as a fuel break and slowing the intensity of a potential fire. Thus, the Roses fire emphasises how a knowledgeable firefighting force transformed a potentially damaging wildfire into one which could be managed in a manner that avoided direct losses, whilst also increasing the overall future resilience of the landscape.

### ASK military site, the Netherlands

The artillery shooting range ASK in ‘t Harde, The Netherlands, is a 5400 ha Department of Defence site where wildfires are inevitable and the landscape is designed with this in mind. The site primarily consists of heathland (*Calluna vulgaris*), Molinia grass and woodland, including coniferous and deciduous species, on nutrient poor soils. Because of year-round shooting practice, the ASK has a high fire risk, as fires are known to occur with ammunition (Short and Finney 2022).

Fires are a known risk within this landscape (Theme 1), however several strategies are utilised to ensure they remain low intensity. Management of the site to counter wildfire risks was mostly triggered after the area experienced a large and damaging wildfire in 1970. ASK’s landscape management is overseen by the land manager, whilst fire risk is quantified and managed by the fire chief; their relationship is based on mutual understanding and trust (subtheme 3.3). Outside fire events, the land manager is in charge of site management; when a fire ignites, the fire chief steps in. There is an extensive collaboration agreement surrounding fire risk, resulting in an integrated fire management covenant covering a (1) description of the terrain and its activities; (2) fire prevention plan including fuel management, fuel breaks, access routes, and fire use; and (3) operational fire suppression plan, including support arrangements with the regional and national civilian fire service (subthemes 3.2, 4.2). The covenant ensures that responsibilities and duties surrounding fire risk are quantified prior to an event, alongside producing accurate risk perceptions of different site locations (subtheme 3.1). Whilst ASK does not have typical communities in the sense of villages or towns inside their site, the different actors involved in maintaining and using the site offer a similar dynamic to a community. The success of the collaboration between the managers, an ongoing process, is a significant factor in the smooth facilitation of fire mitigation strategies.

In terms of fire and keeping the landscape open for military use, the main factors considered by the land manager and fire chief when designing the landscape is what they can do for biodiversity, and what they need to do for fire safety. A toolbox of methods are used, including controlled burning (subtheme 2.2) and accessing subsidies. The site has been categorised using a landscape ecological system analysis (LESA), whereby the geomorphology, geology, climate, microclimate and cultural history of the area are considered on a 1km^2^ scale. From the LESA, the site is split into six zones, whereby each zone has a different goal and associated management strategies. This highly managed landscape has produced a patchwork mosaic of species and land cover (subtheme 2.1), which can reduce fire spread whilst also promoting high biodiversity.

When a fire does occur, there is a high response capacity. The firefighters are well trained to deal with wildfires, and during shooting practice ignitions are anticipated in a safe zone at 0.5–1 km distance (depending on fire danger), ensuring short travel times and rapid initial attack (subtheme 4.2). In some areas the fire may be allowed to burn at a low intensity, depending on the nature present within that zone. The land manager may be present at fires to guide these burns and instruct what should and should not be burnt, with biodiversity in mind. The rapid response to these events ensures that, for the majority of the time, there are no great losses from these fires (theme 4) and the vegetation recovers rapidly (subtheme 5.1). Afterwards, the managers learn from each large event by collecting photos and data, alongside making joint evaluations (subtheme 3.1).

ASK’s adherence to the common themes and proposed definition of a resilient landscape, whilst maintaining its function as a military site and biodiversity hub, encapsulates how to live with fire as a feature of the landscape. Quantifying the fire risk and adapting the capacity of response measures accordingly, alongside strategic management, has worked effectively to increase resilience of the landscape. This example emphasises what can be achieved when awareness and actions surrounding wildfire events are woven into everyday decision making and long term proactive strategies, rather than responses being solely reactive.

### Linking fire resilient landscapes in science and practice

Exploring these practical case studies emphasises the value of applying the common themes of a fire resilient landscape, illustrating how the themes and proposed definition can be used as a tool to critically evaluate landscapes on different scales. These perspectives can also be utilised to highlight any positive feedbacks between the themes. The positive feedbacks signify that concentrating on a singular aspect is unlikely to be as beneficial in the creation of a fire resilient landscape as looking at the concept from an integrated perspective (Smith et al. 2016). For example, accepting and using fire will help create or maintain a mosaic landscape through the removal of certain fuels or the propagation of diverse species (Alcasena et al. 2019; Badia et al. 2019), as shown in the Roses fire. However, for fire to be accepted, stakeholder engagement and trust is essential (Sharp et al. 2013), emphasised within the ASK site and the mutual respect the site managers have for each other. Maintenance of a mosaic landscape has positive impacts for suppression efforts in the future; offering firefighters safe spaces and slowing fire intensity and rate of spread (Castellnou et al. 2019). The presence of firefighters with experience and knowledge of wildfire events, as seen in both examples, alongside these safe spaces in which to work, can help avoid significant losses. Avoiding significant loss will help to ensure that the area can recover, returning to a somewhat stable state, fulfilling the original concept of resilience (Holling 1973).

The perception that it is not always desirable to have this stable state as identical before and after the wildfire event moves beyond the basic concept of resilience towards a transformative-resilience approach (McWethy et al. 2019). Transformative-resilience is particularly important when acknowledging the impact that climate change may have on fire regimes and the fact that landscapes are not static entities. What was a resilient landscape previously may need adaptations to continue being resilient in the future (Schoennagel et al. 2017; McWethy et al. 2019; Stoof and Kettridge 2022). It is also significant to acknowledge this concept within the five themes of resilience explored in this paper. For a landscape to remain resilient, the proportion and relevance of each theme may change with time. To address the concept of a fire resilient landscape in a manner which adapts to change whilst also adhering to the five themes, it is vital to return to the underlying argument that these interventions must be made holistically, with diversity in disciplines and social backgrounds (Sect. 2; McWethy et al. 2019; Smith et al. 2016; Stoof and Kettridge 2022). Approaching fire resilient landscapes from this integrated perspective can help bridge the gap between science and practice, as well as improving the social diversity of those involved in fire, strengthening the design, initiation and maintenance of fire resilient landscapes.

## Conclusions

The concept of a fire resilient landscape is often re-defined according to the aim of a particular research question, with the singular definition found (Magalhães et al. 2021) covering only ecological characteristics of a landscape. We recognise that a landscape also comprises of social processes, such as the ancestral use of fire to open and maintain particular ecosystems. We engaged with the wider fire community to extract the five common themes and eleven subthemes of a fire resilient landscape, as follows:

- acceptance and use of fire (fire as a natural process—prescribed burning)
- landscape management (mosaic landscape—fuel management)
- community engagement (knowledge sharing, collaboration, education—trust—prevention)
- loss avoidance (absence of long lasting damage—trained suppression force)
- recovery (return to stable state—fire resilient species)

We utilised these themes to propose an integrated definition: ‘*A fire resilient landscape is a socio-ecological system that accepts the presence of fire, whilst preventing significant losses through landscape management, community engagement and effective recovery’.* The importance of engaging with the concept holistically is emphasised within this definition and the themes. These common themes, alongside their interconnections, can be applied across a range of spatio-temporal scales. We illustrated this application in case studies in areas of both existing and emerging wildfire risk, and discussed how the positive feedbacks between these themes can assist in creating and maintaining a fire resilient landscape, giving tangible aims for researchers and those working in practice.

## Supplementary Information

Below is the link to the electronic supplementary material.

Supplementary file1 (PDF 108 kb)

## Data Availability

Underyling data for this research can be found open access at: 10.5281/zenodo.8075823.
