# Supplementary material for: What is a fire resilient landscape? Towards an integrated definition
Source: Ambio. 2023 Jun 30;52(10):1592–602. doi: 10.1007/s13280-023-01891-8 (PMC10460754; doi:10.1007/s13280-023-01891-8)
Supplement: Supplementary file 1 — Supplementary file1 (PDF 108 kb) [file 13280_2023_1891_MOESM1_ESM.pdf]

Ambio

Supplementary Information

This supplementary information has not been peer reviewed

Title**: What is a fire resilient landscape? Towards an integrated definition**

Authors: Fiona E. Newman Thacker, Harm Bartholomeus, Marc Castellnou Ribau, Cathelijne R. Stoof

1.0. Literature Review

Literature in the form of research articles were extracted from Web of Science and Scopus for the years 1995 – 2022. Title, key words and abstract were searched for the words ‘Fire’ AND ‘Resilient’ AND ‘Landscape’. The two datasets were amalgamated into one Excel file and the duplicate studies removed based on Abstract. The extracted data was then evaluated using exclusion criteria.

- Focus point of research is not landscape resilience (example: some articles concentrated solely on animal responses).
- Resilience is not directly linked to wildfire (example: looking at resilience to other disturbances, bark beetles, deforestation)

This criteria was used to focus the literature towards the concept of fire resilient landscapes. Initially the title and keywords were analysed using this exclusion criteria to judge whether the article content was related to fire resilient landscapes. If it was not possible to discern the relevance of the article, the cell colour was changed. These articles were then examined by reading through the abstract. Those that were then deemed relevant were changed to a green cell colour, and those that were not were removed. This resulted in 171 articles remaining from an original 263.

2.0. Thematic Analysis

Survey responses were first collated into one document, then separated into the two groups discussed in the manuscript body. Using Braun and Clarke’s (2006) methodology, they were initially coded according to the relevant features within each answer, when looking at the answers from the perspective of the question; ‘How can a fire resilient landscape be defined’. The codes were then grouped into common themes by clustering together those with commonalities. The common themes were then named.

Next, subthemes were found within the common themes. Within this review stage, the common themes were also analysed to ensure their overall fit.

**Common Theme**	**Mentions**	**Subtheme**	**Mentions**	**Total**
Theme 1 – Acceptance and use of fire	**11**	Fire as a natural process	**33**	**58**
		Prescribed burning	**14**	
Theme 2 – management of landscape	**15**	Fuel Management	**23**	**59**
		Mosaic design	**21**	
Theme 3 – community engagement	**21**	Knowledge exchange collaboration & education	**36**	**73**
		Trust	**6**	
		Prevention	**10**	
Theme 4 – loss avoidance	**19**	Absence of damage	**18**	**53**
		Trained suppression force	**16**	
Theme 5 - recovery	**7**	Return to stable state	**21**	**38**
		Fire resilient species	**10**	

Table S1: showing the number of mentions within responses of each theme and subtheme.

Once the themes and subthemes had been derived, we quantified the number of times each theme/sub theme was mentioned within the answers. Most mentions fell within a subtheme of the overall common theme, however there were some elements of the responses which could only be categorised using the common theme. Some answers mentioned the same theme multiple times within their response, these were classified as individual mentions.

2.1. Participant information

Participants originated from 13 different countries, the breakdown of which can be seen in Figure 2.

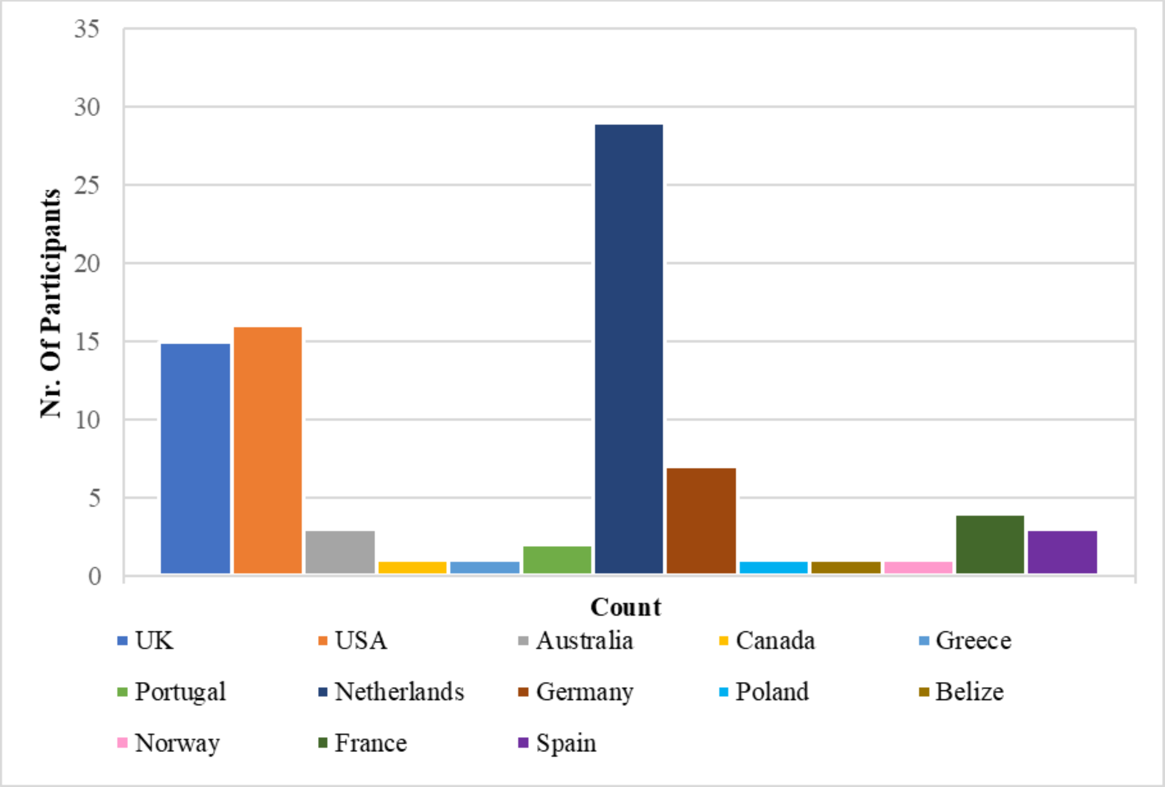

Figure S1: Participant count per country of origin
